# Predictive Value of Cetuximab-Induced Skin Toxicity in Recurrent or Metastatic Squamous Cell Carcinoma of the Head and NECK

**DOI:** 10.3389/fonc.2018.00616

**Published:** 2018-12-13

**Authors:** Shinya Uozumi, Tomohiro Enokida, Shinya Suzuki, Aya Nishizawa, Hayato Kamata, Tomoka Okano, Takao Fujisawa, Yuri Ueda, Susumu Okano, Makoto Tahara, Masakazu Yamaguchi

**Affiliations:** ^1^Department of Pharmacy, National Cancer Center Hospital East, Kashiwa, Japan; ^2^Department of Head and Neck Medical Oncology, National Cancer Center Hospital East, Kashiwa, Japan; ^3^Department of Dermatology, National Defense Medical College Hospital, Tokorozawa, Japan

**Keywords:** skin toxicity, cetuximab, predictive value, head and neck cancer, squamous cell carcinoma, recurrent, metastatic

## Abstract

**Background:** Skin toxicity is a common adverse event during cetuximab (Cmab) treatment. However, few reports have investigated the correlation between skin toxicity and the efficacy of Cmab in patients with recurrent or metastatic squamous cell carcinoma of the head and neck (R/M SCCHN).

**Methods:** We retrospectively reviewed 112 R/M SCCHN patients who received palliative chemotherapy with Cmab. Main eligibility criteria included primary disease in the oral cavity, hypopharynx, nasopharynx, oropharynx, or larynx; no prior history of EGFR-directed therapy; receipt of Cmab plus chemotherapy as first-line therapy for recurrent or metastatic disease; and follow-up for more than 90 days. We analyzed the time to first occurrence and time of maximum grade skin toxicity, and its predictive value with regard to treatment efficacy.

**Results:** After a median follow-up of 393 days (range 109–1501 days), 105 (94%) and 20 (18%) patients had skin toxicity of any grade and grade 3, respectively. Among them, 8 patients with grade 3 acneiform rash, skin rash, or paronychia within 90 days after treatment initiation (“early skin toxicity”) had improved progression-free survival (PFS) (log-rank test, *P* = 0.045; 2-year PFS, 25.0 vs. 2.9%) and overall survival (OS) (log-rank test, *P* = 0.023, 2-year OS, 50.0 vs. 14.4%) compared with those with < grade 3 toxicity. A greater proportion of patients with early skin toxicity than patients without this toxicity could proceed with Cmab maintenance (88 vs. 44%, *P* = 0.021). Multivariate analysis identified early skin toxicity as an independent predictor of better PFS (hazard ratio [HR] = 0.363, 95% confidence interval [CI] 0.142–0.924, *P* = 0.034) and OS (HR = 0.187, 95% CI: 0.045–0.781, *P* = 0.022).

**Conclusion:** Grade 3 Cmab-induced skin toxicity within 90 days was associated with better survival in R/M SCCHN. Effective rash management therefore seems necessary to realize the benefit of Cmab treatment.

## Introduction

Head and neck cancer is the sixth-most common cancer, and more than 600,000 new cases are diagnosed annually worldwide (1, 2). In Japan, approximately 20,000 new cases are diagnosed annually (3). Despite optimal treatment, locoregional recurrence will occur in 60% of these patients, often in irradiated areas, and distant metastasis will develop in 20%. The prognosis of patients with recurrent or metastatic disease is poor and their therapeutic options are limited, with most requiring palliative chemotherapy.

Cetuximab (Cmab) is an epidermal growth factor receptor (EGFR) inhibitor which plays an important role in epithelial malignancies, including squamous cell carcinoma of the head and neck (SCCHN). The phase III EXTREME trial reported that the addition of Cmab to platinum/5FU significantly improved overall survival (OS), progression-free survival (PFS) and response compared with platinum/5FU in first-line treatment of patients with recurrent or metastatic squamous cell carcinoma of the head and neck (R/M SCCHN) (4). Accordingly, the regimen has been recognized as a standard care for the disease worldwide, including Japan. One of its major side effects is skin toxicity, manifesting as a skin rash, acneiform rash, paronychia, dry skin, hair growth disorders, pruritus, or nail changes. Studies in multiple malignancies have shown that there is no apparent difference in the incidence or severity of Cmab-induced skin toxicity between races, while, the occurrence of more severe Cmab-induced skin toxicity correlates with better treatment response and longer survival (5–15). However, this correlation of Cmab-induced skin toxicity with efficacy has not been shown for R/M SCCHN. Here, we examined whether Cmab-induced skin toxicity predicts treatment efficacy in patients with R/M SCCHN.

## Methods and Materials

We have reviewed the medical records of R/M SCCHN patients who received palliative chemotherapy with Cmab in various combination (5-FU + cisplatin; CDDP or carboplatin: CBDCA + Cmab, paclitaxel: PTX + CBDCA + Cmab and PTX + Cmab) at the National Cancer Center Hospital East Japan between December, 2012 and December, 2016 (Table 1). Main eligibility criteria were age ≥20 years; primary disease in the oral cavity, hypopharynx, nasopharynx, oropharynx, or larynx; no prior history of EGFR-directed therapy; receipt of chemotherapy plus Cmab as first-line therapy for recurrent or metastatic disease; and follow-up for more than 90 days. All patients received Cmab at a dose of 400 mg/m^2^ IV on day 1 and 250 mg/m^2^ weekly thereafter. In the Cmab plus platinum agent (cisplatin or carboplatin) group, patients who had at least stable disease received Cmab monotherapy (maintenance therapy) until disease progression or until unacceptable toxic effects occurred after a maximum of six cycles of platinum administration. In the paclitaxel and Cmab group, patients received these agents until paclitaxel-induced toxic effects became unacceptable, after which they continued with Cmab maintenance until disease progression occurred. The patients were not included in a consecutive way. In accordance with the MASCC guidelines (16), we used prophylactic therapy for Cmab-induced skin toxicity, consisting of a skin moisturizer (heparinoid lotion) applied to the body and face twice a day, and oral minocycline 100 mg twice a day, which was started at the beginning of the Cmab-containing regimen. In addition, topical steroids were initiated after the emergence of any skin toxicities. Difluprednate (very strong) 0.05% and hydrocortisone butyrate (mild) 0.1% were applied to the body and face, respectively. The study was approved by the Clinical Research and Ethical Review Board of our institution (task number: 2016-229).

**Table 1:** Patient characteristics (*n* = 112).

**Characteristic**	
**Age (years)**	
Median (range)	64 (26–78)
**Sex**, ***n*** **(%)**	
Male	94 (84)
Female	18 (16)
**ECOG PS**, ***n*** **(%)**	
0/1/2	54 (48)/54 (48)/4 (4)
**Primary site**, ***n*** **(%)**	
Oral cavity	39 (35)
Hypopharynx	33 (29)
Nasopharynx	15 (13)
Oropharynx	12 (11)
Larynx	13 (12)
**Treatment regimen**, ***n*** **(%)**	
5-FU + CDDP or CBDCA + Cmab	33 (30)
PTX + CBDCA + Cmab	36 (32)
PTX + Cmab	43 (38)

*ECOG PS, Eastern Cooperative Oncology Group performance status; 5FU, 5-fluorouracil; CDDP, Cisplatin; CBDCA, Carboplatin; Cmab, Cetuximab; PTX, Paclitaxel*.

### Skin Toxicity Evaluation and Grading

The Cmab-induced skin toxicity was evaluated and graded using the Common Toxicity Criteria for Adverse Events (CTCAE version 4.0) by the same medical oncologist in charge per patient throughout the treatment. The dermatologist (NA) and registered pharmacist (US) supervised and supported the evaluation to share the same criteria and to reduce inconsistency in observation.

### General Principles of Cmab Interruption and Reintroduction

When the grade 3 or worse skin toxicities were observed at the day of Cmab administration, physician omitted Cmab at least one week, and restarted it after the toxicity recovered to Grade 2 or less. In addition, if it is judged that trend of exacerbation was apparent, physician could skip Cmab even in the case of grade 2 skin toxicity, and restarted it as soon as the toxicity recovered to acceptable Grade 2 or less. For patients who experienced Cmab interruption, additional medications (e.g., oral antihistamine and antibiotics, topical antibiotics and a higher-potency topical steroid) were considered at a physician's and dermatologist's discretion. Additionally, when Cmab interruption continued even though the additional medication was given, dose reduction of Cmab could be applied (e.g. dose level 0: 250mg/m^2^, dose level−1: 200mg/m^2^, dose level−2: 150mg/m^2^). In case that further dose reduction is required after doses of cetuximab reduced by 2 levels, the discontinuation of Cmab was considered.

### Statistical Analysis

We analyzed the time to first occurrence and time of maximum grade skin toxicity and its predictive value with regard to treatment efficacy. PFS was defined as the period from the commencement of treatment to the date of confirmation of disease progression or death. OS was determined as the period from the commencement of treatment to the date of death from any cause or the date of the last follow-up. PFS and OS were calculated by the Kaplan–Meier product-limit method. The landmark-time analysis was applied to PFS and OS counted from 90 days after the start of therapy. Hazard ratios (HRs) were calculated by Cox regression analysis. Univariate analyses were undertaken to evaluate the relationship between the pretreatment clinical variables and the risk of development of skin toxicity using the χ2 test or Fisher's exact test. Multivariate analysis was undertaken using logistic regression to identify significant factors associated with PFS and OS. We used SPSS software (version 17.00, SPSS, Inc., Chicago, IL, USA) for the statistical analysis. *P* < 0.05 were considered to indicate statistical significance.

## Results Patient Characteristics

**Figure 1:**
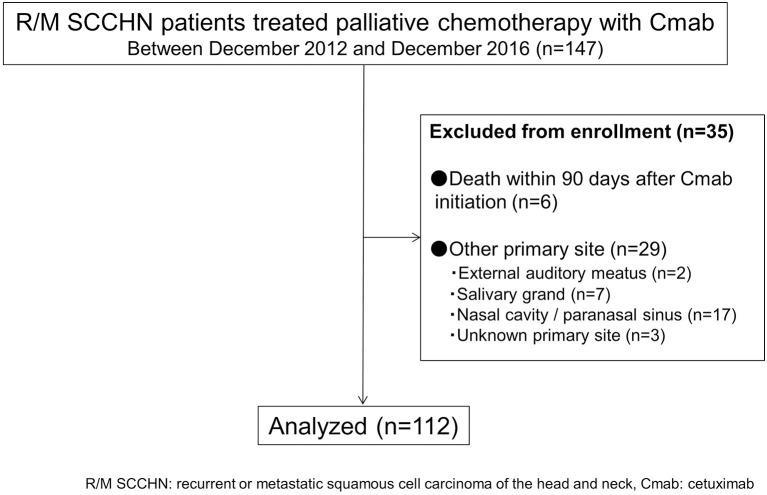
Flow diagram of patient inclusion.

A total of 112 cases were available for analysis (Figure 1). Most patients were men (84%) with a median age of 64 years (range 26–78 years). The main primary disease sites were the oral cavity (35%) and hypopharynx (29%). A total of 33patients (30%) received 5-FU + CDDP or carboplatin: CBDCA + Cmab, while 36 patients (32%) received PTX + CBDCA + Cmab. All other patients were treated with a combination of Cmab and paclitaxel (Table 1).

### Incidence and Characteristics of Cetuximab-Induced Skin Toxicity

After a median follow-up of 393 days (range 109–1501 days), 105 patients (94%) experienced Cmab-induced skin toxicity. Although no grade 4 toxicity was observed, 20 patients (18%) developed skin toxicity of grade 3. Among these, 8 patients (40%) experienced grade 3 toxicity within 90 days after the start of treatment (Table 2).

**Table 2:** Cmab-induced skin toxicity (*n* = 112).

	**Grade 1**		**Grade 2**		**Grade 3**		**All Grades**	
	**≤90days**	**Overall**	**≤90days**	**Overall**	**≤90days**	**Overall**	**≤90days**	**Overall**
Acneiform rash	17 (15)	24 (21)	40 (36)	54 (48)	5 (4)	9 (8)	62 (55)	87 (78)
Paronychia	13 (12)	24 (21)	11 (10)	26 (23)	1 (1)	6 (5)	25 (22)	56 (50)
Skin rash	18 (16)	23 (21)	14 (13)	27 (24)	2 (2)	6 (5)	34 (30)	56 (50)
Fissures	25 (22)	33 (29)	22 (20)	39 (35)	0 (0)	0 (0)	47 (42)	72 (64)
Xerosis	25 (22)	36 (32)	17 (15)	32 (29)	0 (0)	1 (1)	42 (38)	68 (61)
Total with the toxicity*	59 (53)	78 (70)	75 (67)	88 (79)	8 (7)	20 (18)	101 (90)	105 (94)

*Data are presented as n (%)*.

There were no apparent differences in sex, age, primary site, type of combination, treatment regimen, performance status, or body surface area between patients with and without skin toxicities (Table 3.

**Table 3:** Univariate analysis of possible factors related to skin toxicity (≥ Grade 3).

**Variable**	***n***	**≥Grade 3**	**<Grade 3**	***P*-value**
**(A) OVERALL**				
**Age**				
<70	85	16 (19)	69 (81)	0.636
≥70	27	4 (15)	23 (85)	
**Sex**				
Male	94	17 (18)	77 (82)	0.886
Female	18	3 (17)	15 (83)	
**PS**				
0	54	12 (22)	42 (78)	0.244
1 or 2	58	8 (14)	50 (86)	
**BSA (m**^**2**^**)**				
<1.62 (median)	56	7 (12)	49 (88)	0.139
≥1.62 (median)	56	13 (23)	43 (77)	
**Primary site**				
Oral	39	6 (15)	33 (85)	0.618
Non-oral^†^	73	14 (19)	59 (81)	
**Treatment regimen**				
5-FU + CDDP or CBDCA + Cmab	33	8 (24)	25 (76)	0.497
PTX + CBDCA + Cmab	36	6 (17)	30 (83)	
PTX + Cmab	43	6 (14)	37 (86)	
**Type of combination**				
Doublet	43	6 (14)	37 (86)	0.394
Triplet	69	14 (20)	55 (80)	
**(B)** **≤90 DAYS**				
**Age**				
<70	85	6 (7)	79 (93)	0.951
≥70	27	2 (7)	25 (93)	
**Sex**				
Male	94	7 (7)	87 (93)	0.775
Female	18	1 (6)	17 (94)	
**PS**				
0	54	4 (7)	50 (93)	0.916
1 or 2	58	4 (7)	54 (93)	
**BSA (m**^**2**^**)**				
<1.62 (median)	56	2 (4)	54 (96)	0.142
≥1.62 (median)	56	6 (11)	50 (89)	
**Primary site**				
Oral	39	3 (8)	36 (92)	0.869
Non-oral^†^	73	5 (7)	68 (93)	
**Treatment regimen**				
5-FU + CDDP or CBDCA + Cmab	33	4 (12)	29 (88)	0.412
PTX + CBDCA + Cmab	36	2 (6)	34 (94)	
PTX + Cmab	43	2 (5)	41 (95)	
**Type of combination**				
Doublet	43	2 (5)	41 (95)	0.419
Triplet	69	6 (9)	63 (91)	

*Data are presented as n (%). BSA, body surface area*.

† *Hypopharynx, nasopharynx, oropharynx, larynx*.

### Interruption and Discontinuation of Palliative Chemotherapy With Cmab

Chemotherapy with Cmab was interrupted because of Cmab-induced skin toxicity in 33 patients (29%). Among these, 16 of 20 patients with grade 3 skin toxicity (acneiform rash in 6, skin rash in 4, paronychia in 6) and 17 of 88 patients with grade 2 skin toxicity (acneiform rash in 8, skin rash in 4, paronychia in 4 cases and others in 7 cases, with some patients having more than one toxicity). The median cumulative duration of interruption of treatment due to skin toxicity was 14 days (7–56 days). During the interruption, additional oral antihistamine and/or antibiotics were given to 14 patients while additional topical antibiotics and/or a higher-potency topical steroid was given to 25 patients (Table 4). While, 18 cases experienced Cmab dose reduction because that Cmab interruption continued under the additional medication. Consequently, chemotherapy with Cmab was restarted in almost all cases of treatment interruption, except in one case in which chemotherapy was discontinued at the patient's discretion, despite complete resolution of the skin toxicity.

**Table 4:** Interruption and discontinuation due to Cmab-induced skin toxicity.

**(A) INTERRUPTION AND DISCONTINUATION**	
**Interruption, *n* (%)**	
**Median cumulative duration (range)**	**33 (29%)** **14 days (7-56)**
**Discontinuation, n (%)**	**1 (0.8%)**
**(B) ADDITIONAL MANAGEMENT AFTER CMAB INTERRUPTION**	
**Systemic, *n* (%)**	
**Antihistamine**	**11 (33)**
**Antibiotics**	**11 (33)**
**Prednisolone**	**1 (3)**
**Topical, *n* (%)**	
**Escalation of steroid potency**	**12 (36)**
**Antibiotics**	**10 (30)**

### Predictive Value of Cetuximab-Induced Skin Toxicity for OS and PFS

**Figure 2:**
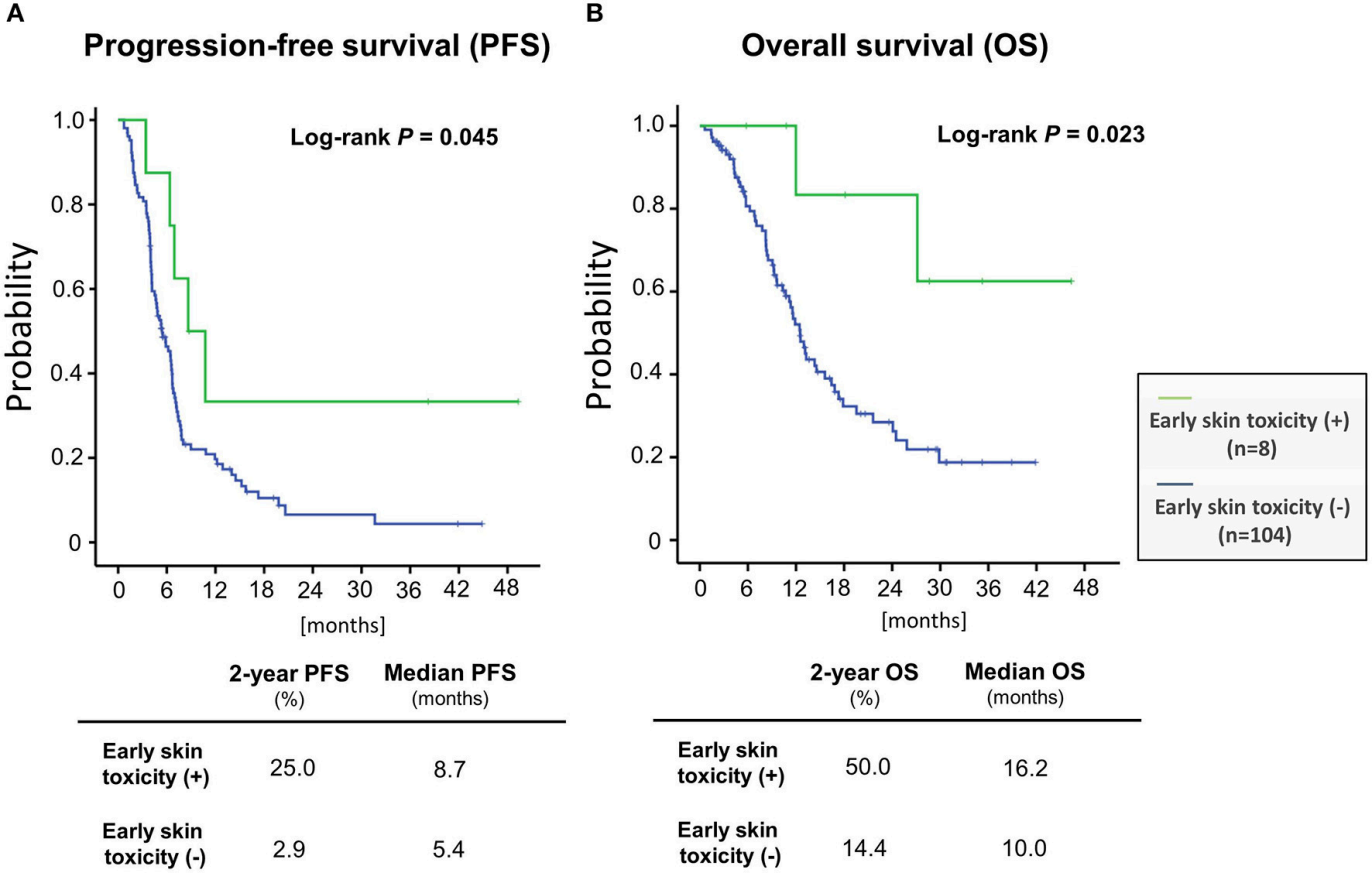
Patient prognoses stratified according to the presence or absence of early skin toxicity **(A)** progression-free survival **(B)** overall survival.

We then examined the correlation between skin toxicity and prognosis. Patients with acneiform rash, skin rash and paronychia of grade 3 severity within 90 days after treatment initiation (“early skin toxicity”) had improved PFS (log-rank test, *P* = 0.045) and OS (log-rank test, *P* = 0.023) compared with those with less than grade 3 toxicity (Figure 2). The 2-year PFS and OS rates of patients with early skin toxicity and those without were 25.0 vs. 2.9%, and 50.0 vs. 14.4%, respectively. Multivariate analysis identified early skin toxicity as an independent favorable prognostic factor for PFS (HR = 0.363, 95% confidence interval [CI] 0.142–0.924, *P* = 0.034) and OS (HR = 0.187, 95% CI 0.045–0.781, *P* = 0.022) (Table 5). Furthermore, a greater proportion of patients with early skin toxicity could proceed with Cmab maintenance than patients without this toxicity (88 vs. 44%, *P* = 0.021) (Table 6).

**Table 5:** Cox regression analysis.

**Variable**	**HR**	**95% CI**	***P*-value**
**(A) PROGRESSION-FREE SURVIVAL**			
Skin toxicity^*^ <Grade 3 ≥Grade 3	Referent 0.363	0.142–0.924	0.034
**Sex** Male Female	Referent 1.819	1.039–3.187	0.036
**Age** <70 ≥70	Referent 0.935	0.574–1.522	0.787
**Primary site** Oral Non-oral^^†^^	Referent 0.983	0.631–1.531	0.938
**Treatment regimen** Doublet Triplet	Referent 0.860	0.565–1.308	0.481
**(B) OVERALL SURVIVAL**			
Skin toxicity^*^ <Grade3 ≥Grade3	Referent 0.187	0.045–0.781	0.022
**Sex** Male Female	Referent 1.135	0.576–2.235	0.715
**Age** <70 ≥70	Referent 1.317	0.751–2.309	0.337
**Primary site** Oral Non-oral^^†^^	Referent 0.554	0.327–0.940	0.028
**Treatment regimen** Doublet Triplet	Referent 0.707	0.416–1.201	0.199

HR, hazard ratio; CI, confidence interval.

* Acneiform rash, skin rash, paronychia.

† *Hypopharynx, nasopharynx, oropharynx, larynx*.

**Table 6:** Cmab maintenance therapy (*n* = 112).

**Maintenance**	***n***	**≥Grade 3, *n* (%)**	**<Grade 3, *n* (%)**	***P*-value**
Yes	53	7 (88)	46 (44)	0.021
No	59	1 (12)	58 (56)	

## Discussion

Several reports have indicated a correlation between the severity of Cmab-induced skin toxicity and treatment efficacy, including a retrospective review of Cmab with radiotherapy for SCCHN that showed a better outcome in patients with a G2-4 rash (5–14). However, few studies have focused on the correlation between Cmab-induced skin toxicity and efficacy in R/M SCCHN. Klinghammer et al. observed a trend toward longer PFS and OS in patients who experienced grade 1 rash compared with those with grade 0 among R/M SCCHN patients who were treated with the combination of Cmab and docetaxel (17). In our present study, we found that severe (≥grade 3) Cmab-induced skin toxicity within 90 days (“early skin toxicity”) is an independent and more robust predictive factor for a favorable clinical outcome after adjusting for sex, age, primary site and treatment regimen (with HR of 0.363 for PFS and HR of 0.187 for OS). Consistent with this finding, patients with early skin toxicity had a better prognosis than that of the entire Cmab plus chemotherapy group in the EXTREME study (2-year OS: 50 vs. 14%) (18). Furthermore, the majority of patients (88%) with early skin toxicity in the current study proceeded to Cmab maintenance therapy, vs. fewer than half of patients (45%) in the Cmab plus chemotherapy group in the EXTREME study. These findings indicate that early skin toxicity is a promising predictor of outcome in treatment with a Cmab-containing regimen in R/M SCCHN.

When considering the significance of skin toxicity as predictor of outcome of treatment with a Cmab-containing regimen, it is important to avoid treatment interruption and discontinuation due to toxicity in order to achieve maximum benefit. However, the current recommendations for the management of Cmab-induced skin toxicity are generally based on expert opinion and consensus (16, 19). In our study, chemotherapy with Cmab was interrupted in 33 patients (29%) because of skin toxicity; however, almost all of those patients were able to restart chemotherapy with Cmab after the addition of an oral antihistamine, oral antibiotics and/or topical antibiotics. Although it is unclear whether this management was appropriate, these treatments might have enabled continuation of the Cmab-containing regimen. However, one patient discontinued chemotherapy because of skin toxicity, even though the toxicity completely resolved. Cmab-induced skin toxicities, especially rash, paronychia and skin fissures, often compromise quality of life and cause psychological discomfort. A multidisciplinary team comprising medical oncologists, dermatologists, pharmacists and nurses needs to be actively engaged in the management of Cmab-induced skin toxicities. A prospective study is also necessary to investigate and standardize the management of Cmab-induced skin toxicities.

Recently, there has been a focus on identification of patients with increased risk of developing EGFR inhibitor-induced rash. At the basic research phase of SCCHN, an EGFR-R521K genotype (G/G) was reportedly associated with increased Cmab-induced skin toxicity (20). Other reports, which included SCCHN patients, found a significant inverse correlation between the plasma concentration of hepatocyte growth factor and EGFR inhibitor-induced rash (17). On the other hand, identification of clinical factors related to the occurrence of Cmab-induced skin toxicity in SCCHN is still lacking, and we were also unable to identify such factors in the present study (Table 3). Men and younger patients with colorectal cancer are considered to be at greater risk of severe Cmab-induced rash (15), but skin toxicity also warrants careful attention in all SCCHN patients who receive Cmab.

## Conclusions

Our present analysis suggested that the occurrence of ≥ grade 3 Cmab-induced skin toxicity within 90 days after the initiation of Cmab was associated with a better prognosis in R/M SCCHN. At the moment, we do not have sufficient clinical knowledge to predict the occurrence of the sing beforehand, which may reflect a different immune status of the patients. However, it is likely important to avoid delays or discontinuation of Cmab, particularly in patients with rapid skin reaction, considering that Cmab appears to play an important role as the mainstay of treatment in this population.

## Author Contributions

SU and TE participated in the study concept and design, interpreted the data, and drafted the manuscript. SS, TF, and SO participated in the study concept and design and interpreted the data. MT extracted, managed, and analyzed the data. All authors provided critical revisions and approved the final manuscript.

### Conflict of Interest Statement

MT and SO receive honoraria from Merck Serono. The remaining authors declare that the research was conducted in the absence of any commercial or financial relationships that could be construed as a potential conflict of interest.
